# Siglec-7 inhibits TLR3-induced pro-inflammatory cytokine production from human monocytes and macrophages

**DOI:** 10.3389/fimmu.2026.1764343

**Published:** 2026-03-17

**Authors:** Justin N. Keeney, Janina Schwarte, Bo Yang, Hendrik Wesseling, Bailin Zhang, Andrew J. McKnight, Subramanya Hegde, Guoxing Wang

**Affiliations:** 1 Immunology & Inflammation Research, Sanofi R&D, Cambridge, MA, United States; 2 Sanofi Global Postdoctoral Fellowship Program, Cambridge, MA, United States; 3 Target, Disease & Systems Biology, Sanofi R&D, Cambridge, MA, United States; 4 Translational Medicine Unit, Sanofi R&D, Cambridge, MA, United States

**Keywords:** agonism and antagonism, antibody, endolysosome, glycosylation, myeloid cells, pro-inflammatory, Siglec-7, TLR3

## Abstract

Immune checkpoint receptors, including Sialic-acid-binding immunoglobulin-like lectins (Siglecs), are critical regulators of immune homeostasis. Siglecs can serve as negative regulators of Toll-like receptor (TLR) signaling, promoting the resolution of inflammatory signaling through feedback inhibition mechanisms. Previous studies demonstrated that Siglec-E, the murine homolog of the human inhibitory receptor Siglec-7, negatively regulates TLR4 signaling by controlling receptor endocytosis. This regulatory mechanism suggests that Siglec-7 may also limit TLR signaling. Here we reveal a novel mechanism whereby Siglec-7 represses endosomal TLR3 activation, compared to other TLRs, in human myeloid cells. Crosslinking Siglec-7 with antibody clone QA79 significantly reduced TNFα secretion in U937 cells, primary monocytes, and macrophages following Poly(I:C) stimulation. Mechanistically, QA79 triggers rapid FcγR-independent internalization and endolysosomal trafficking of surface Siglec-7, which enables the direct co-localization of Siglec-7 with TLR3 within the endolysosome. This co-localization between Siglec-7 and TLR3 suppresses NF-κB phosphorylation, a key pro-inflammatory signaling node downstream of TLR3. These findings establish a previously unrecognized negative regulatory role of Siglec-7 for TLR3-mediated inflammation in myeloid cells, where a disrupted interaction could contribute to autoimmune disease pathogenesis. Targeting this pathway represents a promising therapeutic approach for TLR3-driven autoimmune diseases.

## Introduction

Toll like receptors (TLRs) are critical first-line host defense sensors (1). Disruption of regulatory processes that control TLR activation underpins autoimmune diseases (1). Among these receptors, TLR3 localizes to the endosome and binds to double-strand RNA to induce production of type-1 interferons and pro-inflammatory cytokines (2). Dysregulated TLR3 underlies autoimmune diseases such as Rheumatoid Arthritis, Systemic Lupus Erythematosus, and Type-1 Diabetes (2). Previous reports show Siglec family proteins can negatively regulate TLR signaling with multiple mechanisms described (3–9). Sialic-acid-binding immunoglobulin-like lectins (Siglecs) are evolutionarily conserved type-1 transmembrane proteins found on immune cells that bind to sialic acid residues on glycosylated substrates (10, 11). Multiple Siglecs, such as Siglec-7, can bind to TLR3, however the functional significance of this observation is unknown (12).

The inhibitory immune checkpoint receptor Siglec-7 is constitutively expressed on myeloid and NK cell populations (13). Siglec-7 preferentially binds to glycosylated molecules containing an α2,8, α2,6, or α2,3 terminal-linked sialic acid (14–17). These sialic acids appear on pathogenic substrates such as bacteria (18) and viruses (19–22) as well as non-pathogenic substrates such as zymosan (23) and certain self-ligands (14). Recently, CD43 expressed on T cells was identified as a novel endogenous ligand for Siglec-7 (24, 25). This bidirectional Siglec-7/CD43 crosstalk between APCs and T cells elicits tolerogenic programming of T cells, revealing a Siglec-7-mediated mechanism that negatively regulates T cell activity (26).

Emerging evidence has implicated Siglec-7 in multiple immune-mediated inflammatory conditions including relapsing-remitting Multiple Sclerosis, Type-2 Diabetes, and solid organ transplant rejection (27–29). *In vitro*, antibody-mediated agonism of Siglec-7 decreased inflammatory cytokines from GM‐CSF‐induced eosinophil activation and reduced IgE-mediated cell activation of mast cells and basophils (30–32). Alternatively, antagonist antibody engagement of Siglec-7 enhanced Th1 cytokine synthesis in a mixed leukocyte reaction (26). Siglec-E, the mouse homologue of human Siglec-7, is also implicated in multiple mechanistic autoimmune models (33–36). Siglec-E binds to TLR4 and negatively regulates LPS-induced pro-inflammatory cytokines by promoting TLR4 endocytosis (6). Given the high endogenous expression of Siglec-7 on myeloid cells, it remains unclear what role Siglec-7 plays on monocytes or macrophages in respect to TLR regulation.

Here we show that crosslinking Siglec-7 with antibody clone QA79 selectively reduced TLR3-mediated TNFα production from monocytes and macrophages, but was ineffective in reducing TNFα from other TLRs. Upon antibody binding, Siglec-7 was rapidly internalized in a FcγR independent manner and trafficked to the endolysosome where it co-localized with TLR3 and reduced TLR3-induced NF-κB phosphorylation. Our findings demonstrate a unique inhibitory function of Siglec-7 and could suggest a novel therapeutic mechanism for diseases with over-active myeloid cells that result from endosomal dysfunction.

## Materials and methods

### Reagents

Functional grade mouse IgG1 isotype antibodies specific for Siglec-7 were obtained from eBiosciences (clone QA79) or Biolegend (clone S7.7 or Ultra-LEAF IgG1 isotype control). Functional grade goat anti-mouse F(ab’)2 was obtained from eBioscience.

### Cell culture

#### Cell line

U937 cells were obtained from American Type Culture Collection (ATCC).

#### PBMC Isolation

Leukopaks from healthy donors were obtained from Research Blood Components (RBC). PBMCs were isolated from Leukopaks using a Miletenyi MultiMACs system with a PBMC isolation kit (Miltenyi Biotek) and frozen in C10 Freezing Media (Stem Cell Technologies).

#### Monocytes

Monocytes were isolated from frozen PBMCs using a Monocyte Isolation Kit (Stem Cell Technologies).

#### Macrophages

Monocytes were cultured with 20 ng/mL M-CSF (R&D Systems) containing RPMI media for 3 days prior to conducting a 50% fresh M-CSF media changeout and incubating for another 3 days. Cells were detached with Accutase (Stem Cell Technologies).

All cells were grown in at 37 °C with 5% CO_2_ in RPMI media (10% FBS + 1x Penicillin-Streptomycin).

### Flow cytometry

Whole blood was obtained from Sanofi’s Donor Research Program and red blood cells were lysed with ACK buffer (Gibco). Fc block (Invitrogen) was incubated for 10 minutes before adding the antibody cocktail and stained for 30 minutes. Excess antibody was washed, and cells were incubated in Fixation Buffer (R&D Systems) overnight at 4 °C before analyzing on a Cytek Aurora. The results were plotted and analyzed using FlowJo (BD Biosciences).

U937 cells were stained similarly to whole blood. Antibodies used for profiling can be found in **Supplementary Table 1**.

### Cytokine profiling

2x10^5^ U937 cells, 2x10^5^ monocytes, or 1.5x10^5^ macrophages were blocked using a 5% human serum (Sigma Aldrich) solution on ice for 30 minutes. The indicated concentration of IgG control, QA79, or S7.7 antibodies were diluted in the same buffer and incubated for 30 minutes on ice. Unbound antibody was washed with cold PBS and cells were resuspended in RPMI media containing 2 µg/mL F(ab’)2 and the indicated concentration of TLR ligand (Invivogen). Cells were incubated at 37 °C for 24 hours. Media was collected and frozen at -80 °C until ready for analysis. ELISA-matched antibodies for TNFα and IFNα2 were obtained from Biolegend. TMB substrate was used to detect the peroxidase-labeled secondary antibody and was stopped with Stop Solution (R&D Systems). Absorbance was measured on a SpectraMax M5e (Molecular Devices).

For surface glycan removal, U937 cells were treated with 125 mU Neuraminidase (Sialidase from *Clostridium perfringens*, Millipore Sigma) diluted in pH 6.0 PBS for 30 minutes at 37 °C before blocking and antibody treatment described above.

Additional cytokine profiling was conducted using multiplex assays from Meso Scale Discovery.

### Internalization assay

The assay was described previously (37) and 5 µg/mL QA79 or IgG isotype control, with or without prior incubation with Fc block (Invitrogen, Cat 14-9161-73), were preincubation was described above. Briefly, 1x10^5^ cells were resuspended in warm media before incubating in a 37 °C water bath for the indicated timepoints. Internalization was halted by returning the cells to ice and diluting in cold PBS. Once collected, the cells were stained for 30 minutes on ice. Cells were washed and then fixed in Fixation Buffer (R&D Systems) overnight at 4 °C before analyzing on a Cytek Aurora. The results were plotted and analyzed using FlowJo (BD Biosciences). Reagents used can be found in **Supplementary Table 1**.

### Immunoblotting

5x10^5^ U937 cells were preincubated with 25 µg/mL IgG or QA79 and treated with 1.25 µg/mL Poly(I:C) LMW and 2 µg/mL F(ab’)2 as described above. Once collected, cells were lysed with RIPA buffer containing protease and phosphatase inhibitors (Invitrogen). Lysate was quantified using a BCA assay kit (Thermofisher) and ran on precast polyacrylamide gradient gels (Bio-Rad) before transferring to nitrocellulose membranes with an iBlot2 transfer system (Invitrogen). Membranes were blocked with Starting Block buffer (Invitrogen) and incubated with primary antibodies overnight at 4 °C. Membranes were washed and incubated with HRP-conjugated secondary antibodies (Jackson ImmunoResearch) before developing with SuperSignal West Pico PLUS Chemiluminescent Substrate (Invitrogen) and imaged on a Bio-Rad ChemiDoc MP Imaging System. Densitometry measurements were analyzed in Image Lab 6.1.

Phosphorylated antibodies were striped using Restore PLUS Western Blot Stripping Buffer (Invitrogen) before proceeding as described above. Antibodies can be found in **Supplementary Table 1**.

### scRNAseq

Siglec-7 mRNA levels in human PBMCs were taken from the BLUEPRINT dataset (https://projects.ensembl.org/blueprint/) accessed through Omicsoft Array Studio V12.8.0.15 (Qiagen), as described previously (38).

### Proteomics

Macrophages were differentiated as described above. One-day prior to harvest, 24-well plates were coated with 15 µg/mL QA79 or IgG isotype control. Plates were washed and blocked with RPMI media for 1 hour at 37 °C. 2x10^5^ cells per well were plated and incubated for 3 hours. Cells were subsequently washed with PBS and frozen at −80 °C.

Proteomic sample preparation was performed using the single-pot, solid-phase-enhanced sample preparation (SP3) workflow as previously described (39). Briefly, cells were lysed in lysis buffer containing 2% SDS, 300 mM triethylammonium bicarbonate (TEAB), 300 mM NaCl, 8 M Urea, and protease/phosphatase inhibitors. Lysates were sonicated, clarified by centrifugation (21000 g, 10 minutes, 4 °C), and equal protein amounts were reduced and alkylated with 20 mM tris(2-carboxyethyl)phosphine and 40 mM 2-chloroacetamide at 60 °C for 30 minutes. Cytiva Sera-Mag hydrophilic/hydrophobic beads (1:1) were added at a 10:1 (w/w) bead-to-protein ratio, and SP3 wash steps were carried out according to the published protocol. Proteins bound to beads were digested overnight with trypsin (1:50 w/w; Promega) in 50 mM TEAB. Peptides were eluted using LC-MS grade water and 200 mM TEAB, vacuum-dried, acidified, and loaded onto Evotips according to the manufacturer’s instructions.

Mass spectrometry was performed on a timsTOF HT (Bruker) coupled to an Evosep One liquid chromatography system via a CaptiveSpray nano-electrospray source. Peptides were separated on a 15 cm × 75 µm ID IonOpticks Aurora C18 column (1.6 µm particles) with an integrated CaptiveSpray emitter using the 20-samples-per-day (20 SPD) Whisper method. diaPASEF acquisition was carried out using twenty-one precursor double windows of 25 Da, covering 475–1000 m/z and 1/K0 values of 0.85–1.27 V·s/cm². Raw data were processed by library-free direct DIA+ analysis in Spectronaut v19.8 using the SwissProt human proteome database (January 2022). Search parameters included trypsin specificity with up to two missed cleavages, fixed carbamidomethylation (C), variable methionine oxidation, and a precursor-level FDR cutoff of 1% (Q-value < 0.01). Protein abundances were globally normalized using in-house R scripts. Missing values were imputed using k-nearest neighbors (kNN), and ComBat batch correction was applied to reduce clustering driven by donor batches.

Differential expression data (**Supplementary Table 2**) was filtered to only include proteins with 4 or more identified peptides and a -log(pValue) above 1.3. Protein enrichment analysis was conducted with STRING version 12.0 (https://string-db.org) and graphed in GraphPad Prism.

### Endolysosome co-localization

1x10^6^ U937 cells were blocked prior to incubating with 40 µg/mL IgG or QA79 and treated with 1.25 µg/mL Poly(I:C) LMW as described above. Once collected, cells were incubated with Fc block and viability dye for 10 minutes before staining with anti-Siglec-7-PE for 30 minutes on ice. Intracellular markers were stained using a FoxP3 staining kit (Invitrogen). Stained cells were analyzed on a Cytek Amnis ImageStream Mk II. Quantification of internalized Siglec-7 and co-localization with TLR3 was calculated using IDEAs v6.3. IDEAS measures receptor internalization by comparing fluorescence within a whole-cell mask to an internal-cell mask that excludes the membrane region and focuses on intracellular fluorescent signal. (https://cytekbio.com/pages/imagestream).

Antibodies can be found in **Supplementary Table 1**. All antibodies were directly conjugated except for anti-TLR3 which was conjugated with a Lightning-Link Conjugation Kit (Abcam).

### Statistical analysis

Statistical analyses were carried out using GraphPad Prism and details can be found in the figure legends. The data presented in this article was considered statistically significant at p ≤ 0.05.

## Results

### Siglec-7 crosslinking reduced TNFα and IFNα2 from activated TLR3 in U937 cells

**Figure 1:**
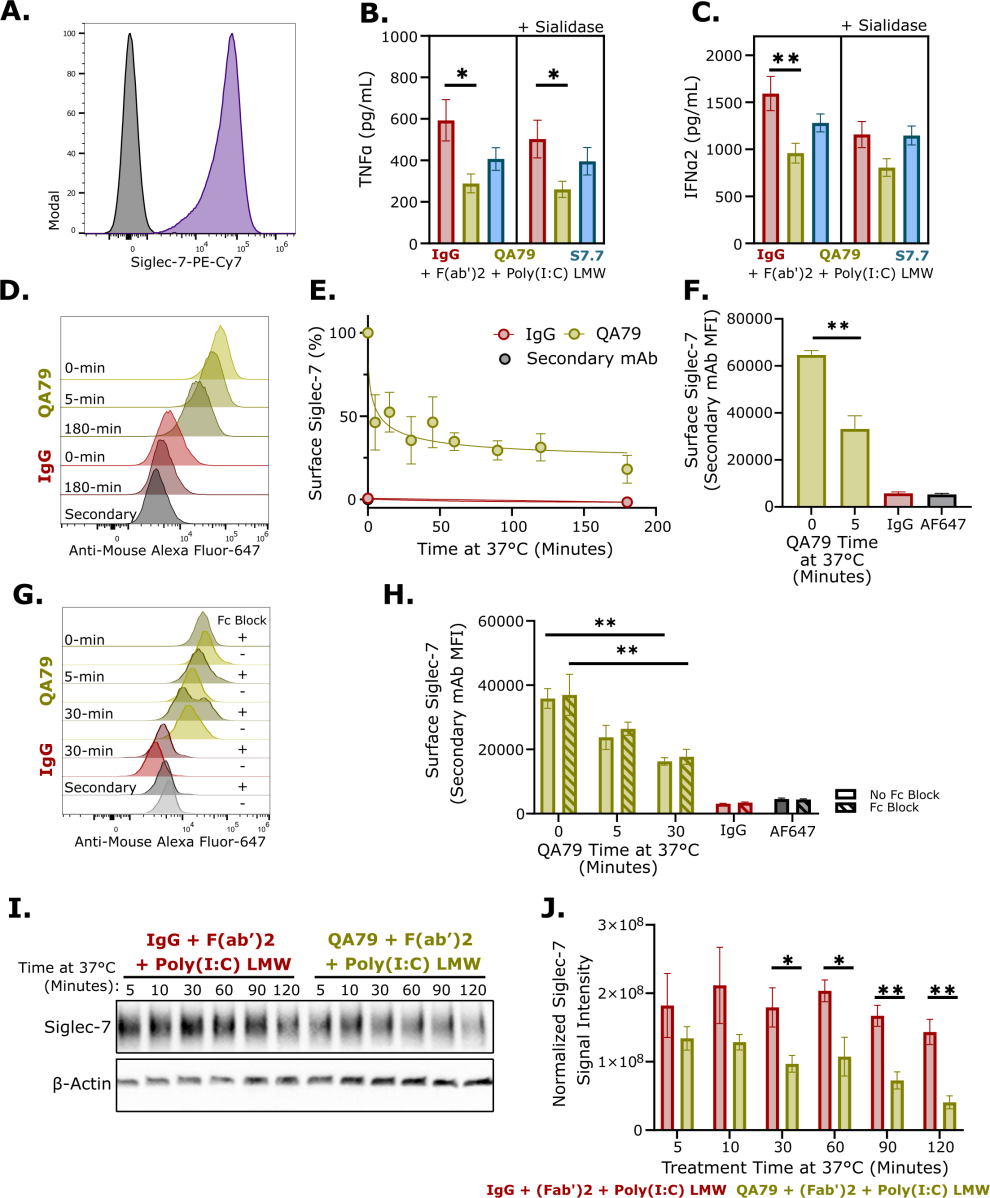
Siglec-7 agonism reduced TLR3-induced pro-inflammatory cytokines and induced rapid internalization of Siglec-7. **(A)** Representative flow cytometry histogram of Siglec-7 staining on U937 cells. Black: Isotype, Purple: Siglec-7 (n=3). TNFα **(B)** or IFNα2 **(C)** ELISA from baseline or 125 mU sialidase treated U937 cells preincubated with 10 µg/mL IgG or QA79 for 30 minutes on ice prior to treating with 2 µg/mL F(ab’)2 and 1.25 µg/mL Poly(I:C) LMW for 24 hours. Data are mean ± SEM, and statistics are a One-Way ANOVA with Tukey’s multiple comparison test; *p<0.05, **p<0.01 (n=4 experiments ran in triplicate). **(D)** Representative histogram of anti-mouse Alexa Fluor-647 staining of U937 cells preincubated with 5 µg/mL IgG or QA79 for 30 minutes on ice prior to incubating at 37 °C for the indicated times. Normalized quantification to time-0 **(E)** or Raw MFI values **(F)** from panel **(D)** Data are mean ± SEM, and statistics are a students T-test; **p<0.01. (n=2 experiments ran in duplicate). **(G)** Representative histogram or **(H)** raw MFI values of anti-mouse Alexa Fluor-647 staining of U937 cells, with or without Fc block, followed by incubated with 5 µg/mL IgG or QA79 for 30 minutes on ice before incubating at 37 °C for the indicated times. Data are mean ± SEM, and statistics are a students T-test; **p<0.01. (n=2 experiments ran in duplicate). Representative western blot **(I)** or normalized Siglec-7 signal intensity **(J)** of U937 cells preincubated with 25 µg/mL IgG or QA79 for 30 minutes on ice prior to incubating at 37 °C for the indicated times (in minutes) cotreated with 2 µg/mL F(ab’)2 and 1.25 µg/mL Poly(I:C) LMW. Data are mean ± SEM, and statistics are a student’s T-test; *p<0.05, **p<0.01 (n=4).

Siglec proteins are known to negatively regulate TLR function, preventing excessive inflammatory signaling (3–8). However, the specific role of myeloid expressed Siglec-7 in regulating TLR-induced cytokine production remains poorly understood. To address this knowledge gap, we used the human monocytic U937 cell line, which exhibited high endogenous Siglec-7 and TLR3 expression (**Figure 1A**; **Supplementary Figure 1A**). Two Siglec-7 antibody clones with distinct functions were obtained: an agonist clone QA79 (30, 31) and antagonist clone S7.7 (26, 40, 41). TLR3 was the initial focus due to the strong binding preference of Siglec-7 for TLR3 (12). QA79-mediated Siglec-7 crosslinking, unlike S7.7, significantly reduced both TNFα and IFNα2 in U937 cells stimulated with Poly(I:C) LMW (**Figures 1B, C**). Poly(I:C) HMW, another TLR3 ligand, showed a similar reduction in TNFα but a significant increase in IFNα2 upon Siglec-7 crosslinking (**Supplementary Figures 1B, C**). Although *cis*-ligands typically mask Siglec-7 and impair downstream signaling (42), removal of cell surface glycans with sialidase did not abolish QA79’s inhibitory activity. QA79 maintained a significant inhibitory effect on Poly(I:C) LMW-induced TNFα production following sialidase treatment (**Figure 1B**). However, QA79 showed only a trending reduction of IFNα2 compared to IgG control after sialidase treatment (**Figure 1C**). We observed reduced IFNα2 baseline levels in the IgG control group after sialidase treatment compared to non-sialidase treated samples, which may account for the non-significant difference in IFNα2 between QA79 and IgG control conditions after sialidase treatment (**Figure 1C**). These findings suggest that QA79 is a stronger agonist antibody than S7.7 after F(ab’)2 crosslinking for Siglec-7-mediated inhibition of TLR3 activation.

Next, we investigated whether Siglec-7 crosslinking could inhibit TNFα produced from other TLRs. Crosslinking Siglec-7 with QA79 did not significantly reduce TLR4-induced TNFα after LPS treatment (**Supplementary Figure 1D**). However, crosslinking Siglec-7 and activating TLR7 and TLR8 with R848 significantly reduced TNFα (**Supplementary Figures 1E, F**). This data showed that crosslinking Siglec-7 with QA79 inhibited endosomal TLR-mediated TNFα production, with the greatest effectiveness against TLR3.

### Antibody engagement induced rapid internalization of Siglec-7

It is understood that inhibitory receptors need to co-localize with activation receptors to inhibit downstream signaling (43). We studied whether cell surface Siglec-7 becomes internalized upon antibody engagement to enable possible co-localization with TLR3. U937 cells were preincubated with QA79 and Siglec-7 surface levels were assessed with a fluorescent secondary antibody. Within approximately 8 minutes, 50% of surface expressed Siglec-7 was internalized (**Figures 1D–F**). After the initial rapid decrease in surface Siglec-7, the amount of surface Siglec-7 steadily reduced over the remaining 3 hours; although at a diminished rate (**Figures 1D–F**).

Since the rapid internalization of Siglec-7 was induced by antibody engagement, we wanted to investigate whether the internalization was Siglec-7 specific or a FcγR-mediated uptake process. To distinguish between these possibilities, we performed an internalization experiment using Fc block prior to QA79 incubation. Fc blockade showed no impact on Siglec-7 internalization after either 5 or 30 minute QA79 treatment compared to non-Fc block treated cells (**Figures 1G, H**), confirming that the observed internalization of Siglec-7 reflects Siglec-7-specific trafficking rather than non-specific FcγR-mediated uptake.

Following receptor internalization, proteins are either recycled or trafficked to endolysosome for degradation (44). To determine the fate of Siglec-7 after internalization, immunoblot analysis was performed. This revealed a significant decrease in total Siglec-7 within 30 minutes of QA79 treatment compared to IgG control (**Figures 1I, J**). This rapid reduction suggests that internalized Siglec-7 undergoes degradation rather than recycling, consistent with a previous QA79-induced degradation study in Ba/F3 overexpression cells (45). Collectively, these findings establish QA79-mediated crosslinking triggers rapid FcγR-independent internalization of surface Siglec-7, providing a mechanistic basis for the reduction in TLR3-induced TNFα in U937 cells.

### Crosslinking Siglec-7 decreased TLR3-mediated TNFα from primary monocytes and macrophages

**Figure 2:**
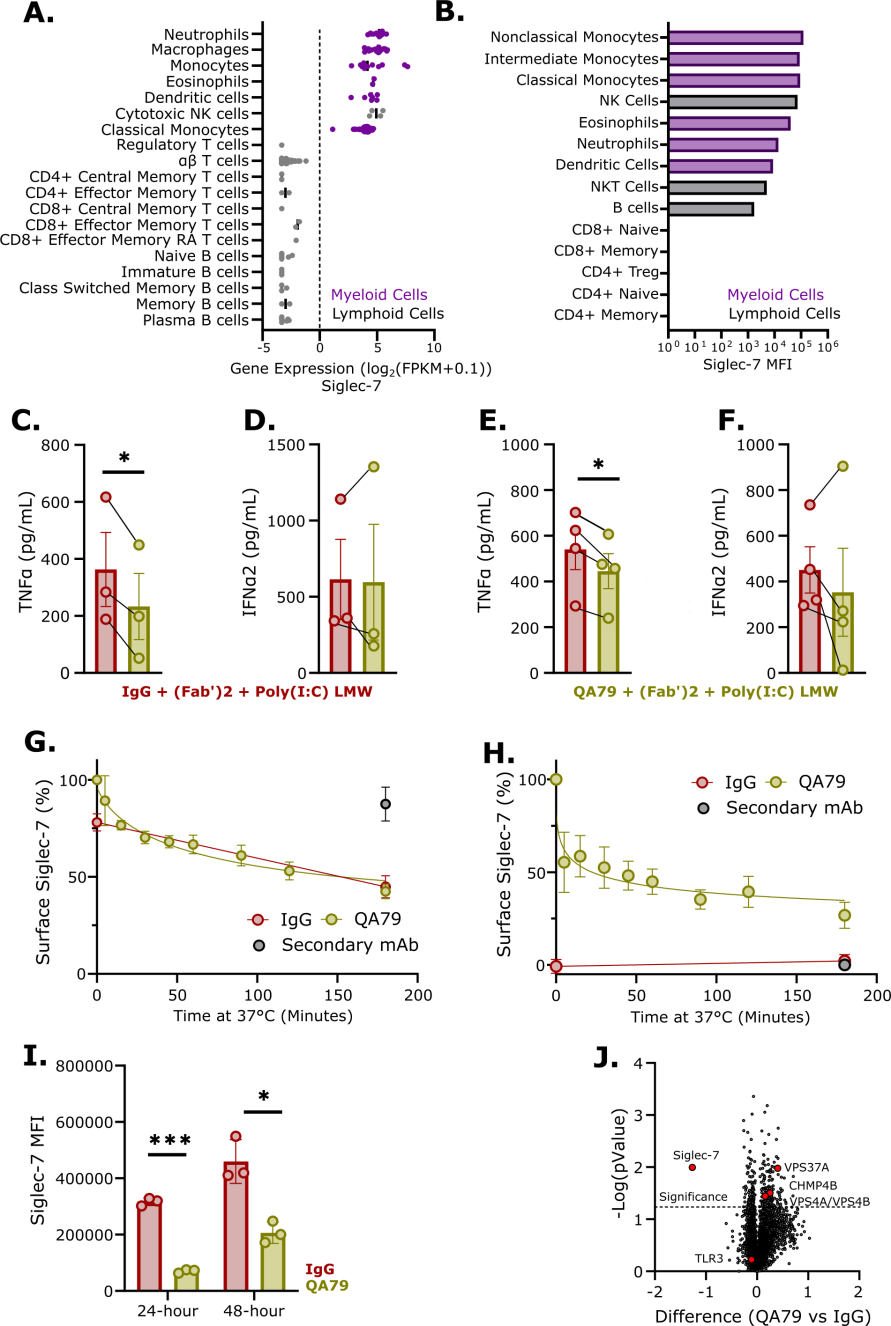
Agonism of Siglec-7 decreased TLR3-mediated TNFα in primary monocytes and macrophages. **(A)** Relative Siglec-7 mRNA expression in human blood immune cells (BLUEPRINT data set). **(B)** Representative Siglec-7 MFI of whole blood immunophenotyping (n=2). TNFα **(C)** or IFNα2 **(D)** ELISA from primary human monocytes preincubated with 20 µg/mL IgG or QA79 for 30-minutes on ice before treating with 2 µg/mL F(ab’)2 and 1.25 µg/mL Poly(I:C) LMW for 24-hours. Data are mean ± SEM, and statistics are a student’s T-test; *p<0.05 (n=3 donors). TNFα **(E)** or IFNα2 **(F)** ELISA from primary human macrophages preincubated with 20 µg/mL IgG or QA79 for 30 minutes on ice before treating with 2 µg/mL F(ab’)2 and 1.25 µg/mL Poly(I:C) LMW for 24 hours. Data are mean ± SEM, and statistics are a student’s T-test; *p<0.05 (n=3 donors). Normalized quantification to time-0 of the anti-mouse Alexa Fluor-647 secondary to detect surface levels of Siglec-7 on primary human monocytes **(G)** or primary human macrophages **(H)** post incubation at 37 °C with 5 µg/mL IgG or QA79. (n=3 donors). **(I)** Flow cytometry profiling of Siglec-7 on primary macrophages after treatment at 37 °C with 5 µg/mL IgG or QA79 for the indicated timepoints. Data are mean ± SEM, and statistics are a student’s T-test; *p<0.05, ***p<0.001 (n=3 donors). **(J)** Volcano plot showing differential total protein abundance in primary macrophages stimulated for 3 hours at 37 °C on plates coated with 15 µg/mL IgG or QA79. (n=3 donors).

Cell lines have distinct immune responses and cytokine profiles compared to primary immune cells due to altered signaling components (46–49). To validate our U937 cell findings in primary cells, we first characterized Siglec-7 expression across human peripheral blood immune cells. Analysis revealed high Siglec-7 expression, at both mRNA and protein levels, on myeloid and NK cell populations (**Figures 2A, B**; **Supplementary Figure 2**). Functionally, QA79-mediated Siglec-7 crosslinking on both monocytes and macrophages reduced TNFα following Poly(I:C) LMW stimulation, while effects on IFNα2 and other pro-inflammatory cytokines were not significantly changed (**Figures 2C–F**; **Supplementary Figures 3A, B**). Additionally, both monocytes and macrophages exhibited rapid internalization of surface Siglec-7 within 5 minutes of QA79 treatment (**Figures 2G, H**). Together these data establish that rapid Siglec-7 internalization and inhibition of TLR3-mediated TNFα production is a conserved feature of myeloid cell biology.

Having confirmed rapid Siglec-7 internalization on primary myeloid cells, we next examined the longer-term fate of Siglec-7 following QA79 engagement. Analysis of Siglec-7 surface levels on macrophages 24 and 48 hours after QA79 treatment showed a persistent and significant reduction (**Figure 2I**). The prolonged reduction of surface Siglec-7 further supports Siglec-7 degradation upon internalization and suggests minimal recycling or *de novo* synthesis within 48 hours.

To further understand the pathways involved in QA79-mediated Siglec-7 internalization, we conducted a proteomic analysis. To induce robust downstream signaling events of Siglec-7, macrophages were treated for 3 hours with plate-bound QA79. Among all proteins in the dataset, Siglec-7 showed the most significant decrease while TLR3 remained unchanged (**Figures 2J**; **Supplementary Table 2**), consistent with our hypothesis of Siglec-7 specific degradation. Protein Set Enrichment analysis revealed QA79 treatment upregulated processes involved in multivesicular body sorting, viral budding, and lysosomal transport (**Figures 2J**; **Supplementary Figure 3C**). This was further confirmed by a significant upregulation of ESCRT pathway components: VPS4A, VPS4B, and VPS37A (**Figure 2J**). These findings suggest a pathway wherein Siglec-7 undergoes receptor-mediated endocytosis with endolysosomal trafficking and degradation. Collectively, our results provided mechanistic insights into the antibody induced internalization of Siglec-7 and demonstrated that QA79-mediated crosslinking of Siglec-7 reduced TLR3-induced TNFα in both primary monocytes and macrophages.

### Siglec-7 is trafficked to the endolysosome after rapid internalization

**Figure 3:**
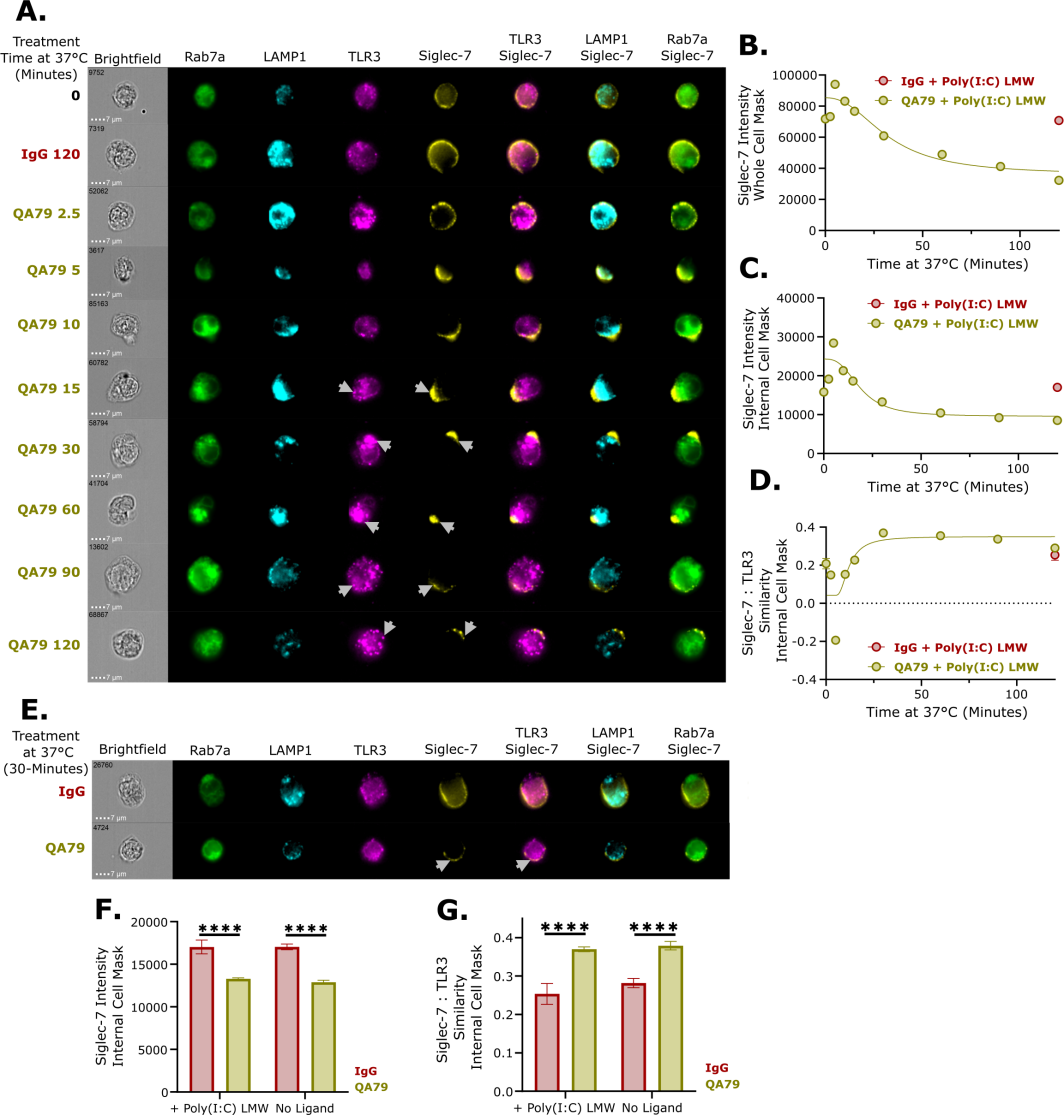
Siglec-7 is trafficked to the endolysosome after rapid internalization. **(A)** Representative ImageStream images of U937 cells treated with 40 µg/mL IgG or QA79 for 30 minutes on ice prior to treating with 1.25 µg/mL Poly(I:C) LMW for the indicated timepoints. Arrows denote regions of signal overlap between Siglec-7 and TLR3. Quantification of Siglec-7 intensity on a whole cell mask **(B)** or internal cell mask **(C)** from the data represented in panel **(A)** Data are mean ± SEM. (n>500 cells per timepoint). **(D)** Quantification of Siglec-7:TLR3 co-localization using the Similarity Intensity index from the internal cell mask of the data represented in panel **(A)** (n>500 cells per timepoint) **(E)** Representative ImageStream images of U937 cells treated with 40 µg/mL IgG or QA79 for 30 minutes on ice prior to incubating at 37 °C for 30 minutes without Poly(I:C) LMW. Arrows denote regions of signal overlap between Siglec-7 and TLR3. Quantification of Siglec-7 Intensity in the internal cell mask **(F)** or Siglec-7:TLR3 co-localization using the Similarity Intensity index from the internal cell mask **(G)**. Data are mean ± SEM, and statistics are a student’s T-test; ****p<0.0001 (n>500 cells per timepoint).

We hypothesized that rapid internalization of surface Siglec-7 facilitates endolysosomal trafficking and subsequent co-localization with TLR3, leading to the reduction in TLR3-mediated TNFα. We used image-based flow cytometry to assess time-dependent Siglec-7 co-localization with TLR3, Rab7a (late endosome marker), and LAMP1 (lysosome marker) in QA79-treated U937 cells (**Figure 3A**). Profiling cell masks generated by the IDEAs software, approximately 50% of Siglec-7 is lost from both cell surface and internal cell masks following QA79 treatment and Poly(I:C) LMW stimulation (**Figures 3B, C**). The images also revealed distinct time-dependent dynamics of Siglec-7 trafficking. Within 5–10 minutes, internalized Siglec-7 rapidly co-localized with Rab7a and LAMP1-positive endolysosome with minimal TLR3 overlap (**Figure 3A**). By 15–30 minutes, TLR3 was recruited to Siglec-7 positive endolysosomes with a peak co-localization between Siglec-7 and TLR3 occurring at 30 minutes (**Figures 3A, D**). Interestingly, co-localization between Siglec-7, TLR3, and LAMP1 did not induce TLR3 degradation (**Figure 3A**).

Next, we evaluated if the co-localization between internalized Siglec-7 and TLR3 is dependent on Poly(I:C) LMW. After 30 minutes of QA79 treatment, there was a comparable reduction of internalized Siglec-7 intensity with or without Poly(I:C) LMW (**Figures 3E, F**). Furthermore, the co-localization of internalized Siglec-7 with TLR3 was similar with or without Poly(I:C) LMW (**Figures 3E, G**). Our data demonstrated that upon antibody engagement, Siglec-7 undergoes rapid endolysosome trafficking, during which it co-localized with TLR3 through a mechanism independent of TLR3 ligand presence.

### Siglec-7 crosslinking inhibited TLR3-mediated NF-κB phosphorylation

**Figure 4:**
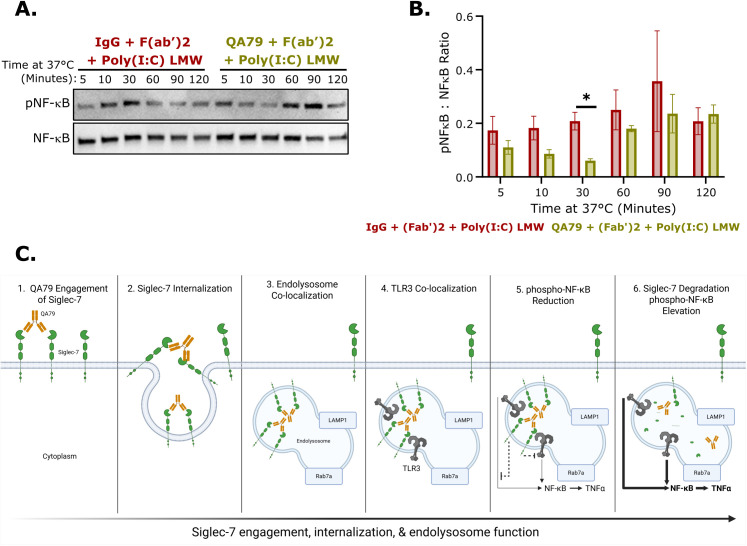
Siglec-7 agonism reduces TLR3-mediated NF-κB phosphorylation. **(A)** Representative western blot analysis and **(B)** quantification of phosphorylated:total NF-κB ratio of U937 cells preincubated with 25 µg/mL IgG or QA79 for 30 minutes on ice prior to incubating at 37 °C for the indicated times cotreated with 2 µg/mL F(ab’)2 and 1.25 µg/mL Poly(I:C) LMW. Data are mean ± SEM, and statistics are a student’s T-test following Grubbs outlier test; *p<0.05 (n ≤ 3). **(C)** Model showing following QA79 treatment, rapidly internalized Siglec-7 co-localized with TLR3 in the endolysosome, leading to a peak reduction in NF-κB phosphorylation, after which Siglec-7 (but not TLR3) undergoes degradation, ultimately resulting in delayed TLR3 signaling and NF-κB phosphorylation begins to recover. Model created with BioRender.com.

TLR3 promotes pro-inflammatory cytokine production primarily through NF-κB activation (2). We next studied whether the co-localization of internalized Siglec-7 with TLR3 after QA79 treatment reduced TLR3-induced NF-κB phosphorylation in U937 cells. Crosslinking Siglec-7 with QA79 decreased NF-κB phosphorylation relative to total NF-κB after Poly(I:C) LMW stimulation with a peak reduction occurring after 30 minutes (**Figures 4A, B**). However, by 60 to 90 minutes the ratio of phosphorylated NF-κB to total NF-κB was not significantly changed between IgG isotype control and QA79 samples (**Figures 4A, B**). This suggests that the loss of Siglec-7 inhibitory function potentially enables restoration of phosphorylated NF-κB at later timepoints.

Collectively, these time-dependent dynamics support a model whereby selective Siglec-7 degradation diminished its inhibitory capacity against TLR3 activation, thereby permitting restoration of TLR3-mediated NF-κB signaling. Overall, QA79-mediated Siglec-7 crosslinking suppressed NF-κB phosphorylation following TLR3 activation, potentially contributing to the diminished TNFα response in U937 cells, monocytes, and macrophages. (**Figure 4C**).

## Discussion

The Siglec family of immune checkpoint receptors play critical roles in maintaining tissue homeostasis with disruption of their regulatory function being implicated in the pathogenesis of multiple disease conditions (50, 51). It is evident that Siglec-7 can play key roles in both oncology and immune-mediated inflammatory disorders. In oncology, Siglec-7 function is widely characterized in multiple tumor microenvironments, particularly with respect to NK cell activity (52–54). Preclinical oncology models show that antibody-mediated antagonism or degradation of Siglec-7 promoted NK cell-mediated cytotoxicity leading to reduced tumor growth and increased survival of Siglec-7 antibody-treated mice in those studies (55–57). Conversely, dysregulated Siglec-7 has been implicated in relapsing-remitting Multiple Sclerosis, Type-2 Diabetes, and solid organ transplant rejection (27–29). *In vitro*, QA79 antibody-mediated Siglec-7 crosslinking reduced GM‐CSF‐induced eosinophil activation and IgE-mediated activation of mast cells and basophils (30–32). In this study, we are the first to show that QA79 antibody-mediated crosslinking of Siglec-7 and not S7.7 significantly inhibited TLR3-induced TNFα in U937 cells (**Figure 1B**), monocytes (**Figure 2C**), and macrophages (**Figure 2E**) compared to other activated TLRs (**Supplementary Figure 1**). We also observed reduced NF-κB phosphorylation (**Figures 4A, B**) following the rapid internalization and endolysosomal trafficking of Siglec-7 where it co-localized with TLR3 (**Figure 3D**).

Protein glycosylation plays critical roles in immune regulation and diminished glycosylation can accelerate autoimmune disease progression (58). Glycan-recognizing receptors require intact ligand-bound glycan structures to initiate bidirectional signaling. Several Siglecs bind to and negatively regulate TLR4-mediated activity (3–9). When sialic acids were removed from TLR4, Siglec-E could no longer bind TLR4 or recruit the inhibitory phosphatases SHP-1 and SHP-2 to suppress TLR4 activation, resulting in enhanced pro-inflammatory cytokine production (7, 59). Disrupting the Siglec-TLR regulatory network may cause or enhance pathological conditions. Future studies should evaluate whether abnormal TLR3 glycosylation could affect the interaction with Siglec-7, potentially dysregulating inflammation-mediated immune responses.

Our study demonstrated that Siglec-7 performs distinct functions depending on its cellular localization. At the cell surface, myeloid-expressed Siglec-7 binds in *trans* to CD43 expressed on T cells (24–26). This Siglec-7:CD43 interaction reduced T cell proliferation and skewed cells toward Th2 cytokine production (26). Supporting this cell surface immunosuppressive role, antibody-mediated antagonism of Siglec-7 (clone S7.7) in mixed leukocyte reactions enhanced Th1 cytokine production and, separately, promoted NK cell-mediated killing of Raji B cells (26, 40). However, Siglec-7 engagement by QA79 on myeloid cells triggered rapid FcγR-independent internalization (**Figures 1D–H**) and endolysosomal trafficking of Siglec-7 (**Figure 3**). While other Siglecs also undergo internalization and endolysosomal trafficking (37, 60–62), our study discovered an unexpectedly rapid antibody-mediated internalization rate of Siglec-7 (clone QA79) on monocytes and macrophages (**Figures 2G, H**). This resembles mouse Siglec-F, another CD33-related Siglec that is internalized independently of clathrin and dynamin and is rapidly trafficked to lysosomes (62). Once Siglec-7 localized to the endolysosome (**Figure 3**), TLR3-mediated TNFα production (**Figure 1B**) and NF-κB phosphorylation was reduced (**Figure 4**). Our data suggests Siglec-7 also inhibits TLR7 and TLR8-mediated TNFα production, though with somewhat lower potency (**Supplementary Figure 1E**). Additional studies should examine if myeloid cells internalize Siglec-7 during immune-mediated inflammatory conditions and how Siglec-7 internalization affects TLR3 signaling.

TLR3 activation produces both pro-inflammatory cytokines and type-1 interferons (2). As such, we also evaluated IFNα2 production following TLR3 activation. While TLR3-mediated TNFα was consistently reduced following QA79 antibody-mediated Siglec-7 crosslinking, IFNα2 reduction was only significantly reduced from U937 cells (**Figure 1C**) as compared to monocytes (**Figure 2D**) and macrophages (**Figure 2F**). This difference likely reflects inherent immune regulatory mechanisms between myeloid cell lines and primary cells (46–49). The differential regulation of TNFα versus IFNα2 by Siglec-7 following QA79 antibody treatment suggests a possible selective mechanism whereby Siglec-7 agonism preferentially inhibits certain specific TLR3 downstream adaptor proteins (63). This selectivity may explain why QA79 crosslinking on U937 cells decreased IFNα2 after TLR3 activation (**Figure 1C**) but slightly increased IFNα2 after TLR7 and TLR8 activation (**Supplementary Figure 1F**) (63). Future mechanistic studies should examine how Siglec-7 crosslinking affects TLR3 downstream adaptor protein recruitment and activation in primary cells.

Most clinical stage immune checkpoint receptor agonist antibodies (*e.g.*, anti-PD1, Peresolimab, Rosnilimab, and anti-BTLA, Venanprubart) target T and B cells (64, 65), yet myeloid-directed approaches may offer advantages in pathologies where myeloid inflammation drives T cell activation. Our data indicates that Siglec-7 may have unexplored therapeutic potential in pathologies driven by TLR3-mediated myeloid dysfunction, such as solid organ transplant rejection. During early allograft rejection, myeloid cells initially infiltrate the transplanted organ and subsequently promote organ recipient T cell migration and activation (29, 66, 67). TLR3 exhibits aberrant activation during acute transplant rejection, while TLR3 knockout mice demonstrate prolonged allograft survival compared to wild-type controls (68–71). Notably, recent clinical findings linked elevated Siglec-7 expression with improved allograft survival (29), suggesting that Siglec-7-mediated regulation of myeloid activation may be protective in this context. Conversely, elevated CD43 expression on CD8+ T cells was associated with decreased allograft survival (72), and inflammation-induced glycan shedding from T cells may disrupt the Siglec-7:CD43 interaction, thereby amplifying inflammatory responses (26, 73).

Autoimmune diseases, like Systemic Lupus Erythematosus (SLE), are also characterized by myeloid dysfunction driven by chronic endosomal TLR activation. In SLE, this stems from anti-nucleic acid autoantibodies forming immune complexes that facilitate self-nucleic acid endosomal uptake and increased type-1 interferon production (74, 75). Supporting a protective role for Siglec-7 in SLE, Siglec-E knockout mice developed SLE-like phenotypes including increased autoantibodies, immune complex deposition, and renal pathology (75). Future studies should investigate the therapeutic potential of targeting Siglec-7 and disrupting TLR3 function in SLE. Collectively, these findings suggest that antibody-mediated Siglec-7 agonism represents a promising strategy to suppress myeloid cell activation during inflammation-driven pathologies, thereby preventing subsequent T cell activation and restoring immune homeostasis.

In conclusion, our results suggest Siglec-7 has unique endolysosome functions. After screening multiple TLRs, we show that Siglec-7 has a previously unrecognized role as an inhibitory receptor for TLR3 in primary human monocytes and macrophages. We demonstrate that Siglec-7 reduced TLR3-mediated TNFα production and NF-κB phosphorylation after treatment with F(ab’)2 crosslinked antibody clone QA79. Moreover, this novel finding provides a strong rationale for future analysis of Siglec-7 function in autoimmune disease conditions and further investigation of whether Siglec-7 agonism can be beneficial to re-balance cellular pathologies caused by aberrant TLR3 signaling.

## Data Availability

The proteomic data have been deposited in Proteome Xchange and jPost Repository under accession numbers PXD075217 - https://proteomecentral.proteomexchange.org/ui?pxid=PXD075217 and JPST004448 - https://repository.jpostdb.org/entry/JPST004448.

## Supplementary material

Supplementary Figure 1 Siglec-7 agonism of U937 cells inhibits proinflammatory cytokines from endosomal TLRs. **(A)** Representative flow cytometry histogram of TLR3 staining in U937 cells. Black: Isotype, Purple: TLR3 (n=2). TNFα **(B)** or IFNα2 **(C)** ELISA from U937 cells preincubated with 10 µg/mL IgG or QA79 for 30 minutes on ice prior to treating with 2 µg/mL F(ab’)2 and 1.25 µg/mL Poly(I:C) HMW for 24 hours. Data are mean ± SEM, and statistics are a Student T-test; *p<0.05 (n=3 experiments ran in triplicate). **(D)** TNFα ELISA from U937 cells preincubated with 10 µg/mL IgG or QA79 for 30 minutes on ice prior to treating with 2 µg/mL F(ab’)2 and 2 ng/mL LPS for 24 hours. Data are mean ± SEM, and statistics are a Student T-test (n=2 experiments ran in triplicate). TNFα **(E)** or IFNα2 **(F)** ELISA from U937 cells preincubated with 10 µg/mL IgG or QA79 for 30 minutes on ice prior to treating with 2 µg/mL F(ab’)2 and 2.5 µg/mL R848 for 24 hours. Data are mean ± SEM, and statistics are a Student T-test; *p<0.05 (n=3 experiments ran in triplicate).

Supplementary Figure 2 Gating strategy of human whole blood Siglec-7 immunophenotyping.

Supplementary Figure 3 Siglec-7 agonism indued minimal cytokine changes in primary cells. MSD multiplex assay profiling additional cytokines known to be downstream of TLR3 activation from primary human monocytes **(A)** or primary human macrophages **(B)** preincubated with 20 µg/mL IgG or QA79 for 30 minutes on ice prior to treating with 2 µg/mL F(ab’)2 and 1.25 µg/mL Poly(I:C) LMW for 24 hours. (n=1 donor ran in triplicate). **(C)** Protein Set Enrichment analysis of proteins significantly altered by a 3 hour stimulation with plate bound QA79 compared to IgG control in primary macrophages. (n=3 donors).

## References

[1] Hosseini AM Majidi J Baradaran B Yousefi M . Toll-like receptors in the pathogenesis of autoimmune diseases. Adv Pharm Bull. (2014) 5:605–14. doi: 10.15171/apb.2015.082, PMID: 26793605 PMC4708030

[2] Hsieh ML Nishizaki D Adashek JJ Kato S Kurzrock R . Toll-like receptor 3: a double-edged sword. biomark Res. (2025) 13:32. doi: 10.1186/s40364-025-00739-5, PMID: 39988665 PMC11849352

[3] Yang D Wu Y Chen G-Y . Regulation of the immune response by Siglec-1 through phosphorylation of Src at Ser17. J Immunol. (2020) 204:68.5–5. doi: 10.4049/jimmunol.204.supp.68.5, PMID: 34749531

[4] Yang D Wu Y Chen G-Y . Siglec-1 negatively regulates TLR4-mediated inflammatory response by uniquely controlling Src phosphorylation at Ser17. J Immunol. (2019) 202:64.17–7. doi: 10.4049/jimmunol.202.supp.64.17, PMID: 34749531

[5] Ishida A Akita K Mori Y Tanida S Toda M Inoue M . Negative regulation of toll-like receptor-4 signaling through the binding of glycosylphosphatidylinositol-anchored glycoprotein, CD14, with the sialic acid-binding lectin, CD33*. J Biol Chem. (2014) 289:25341–50. doi: 10.1074/jbc.m113.523480, PMID: 25059667 PMC4155695

[6] Wu Y Ren D Chen G-Y . Siglec-E negatively regulates the activation of TLR4 by controlling its endocytosis. J Immunol. (2016) 197:3336–47. doi: 10.4049/jimmunol.1600772, PMID: 27619995 PMC5101162

[7] Karmakar J Mandal C . Interplay between sialic acids, siglec-E, and neu1 regulates myD88- and TRIF-dependent pathways for TLR4-activation during leishmania donovani infection. Front Immunol. (2021) 12:626110. doi: 10.3389/fimmu.2021.626110, PMID: 33763070 PMC7982817

[8] Spence S Greene MK Fay F Hams E Saunders SP Hamid U . Targeting Siglecs with a sialic acid–decorated nanoparticle abrogates inflammation. Sci Transl Med. (2015) 7:303ra140. doi: 10.1126/scitranslmed.aab3459, PMID: 26333936

[9] Råberg L Jia F von Mentzer U Venkatakrishnan V Karlsson NG Stubelius A . Sialoglycans modulate Siglec-5-TLR4 interactions in osteoarthritis. iScience. (2025) 28:113979. doi: 10.1016/j.isci.2025.113979, PMID: 41358156 PMC12677102

[10] Reily C Stewart TJ Renfrow MB Novak J . Glycosylation in health and disease. Nat Rev Nephrol. (2019) 15:346–66. doi: 10.1038/s41581-019-0129-4, PMID: 30858582 PMC6590709

[11] Crocker PR Paulson JC Varki A . Siglecs and their roles in the immune system. Nat Rev Immunol. (2007) 7:255–66. doi: 10.1038/nri2056, PMID: 17380156

[12] Chen G-Y Brown NK Wu W Khedri Z Yu H Chen X . Broad and direct interaction between TLR and Siglec families of pattern recognition receptors and its regulation by Neu1. eLife. (2014) 3:e04066. doi: 10.7554/elife.04066, PMID: 25187624 PMC4168287

[13] Nicoll G Ni J Liu D Klenerman P Munday J Dubock S . Identification and characterization of a novel siglec, siglec-7, expressed by human natural killer cells and monocytes*. J Biol Chem. (1999) 274:34089–95. doi: 10.1074/jbc.274.48.34089, PMID: 10567377

[14] Büll C Nason R Sun L Coillie JV Sørensen DM Moons SJ . Probing the binding specificities of human Siglecs by cell-based glycan arrays. Proc Natl Acad Sci. (2021) 118:e2026102118. doi: 10.1073/pnas.2026102118, PMID: 33893239 PMC8092401

[15] Carluccio CD Padilla-Cortés L Tiemblo-Martìn M Gheorghita GR Oliva R Cerofolini L . Insights into siglec-7 binding to gangliosides: NMR protein assignment and the impact of ligand flexibility. Adv Sci. (2025) 12:2415782. doi: 10.1002/advs.202415782, PMID: 40285643 PMC12140324

[16] Crocker PR Blixt O Collins BE van den Nieuwenhof IM Paulson JC . Sialoside specificity of the siglec family assessed using novel multivalent probes IDENTIFICATION OF POTENT INHIBITORS OF MYELIN-ASSOCIATED GLYCOPROTEIN*. J Biol Chem. (2003) 278:31007–19. doi: 10.1074/jbc.m304331200, PMID: 12773526

[17] Yamaji T Teranishi T Alphey MS Crocker PR Hashimoto Y . A small region of the natural killer cell receptor, siglec-7, is responsible for its preferred binding to α2,8-disialyl and branched α2,6-sialyl residues A COMPARISON WITH siglec-9*. J Biol Chem. (2002) 277:6324–32. doi: 10.1074/jbc.m110146200, PMID: 11741958

[18] Avril T Wagner ER Willison HJ Crocker PR . Sialic acid-binding immunoglobulin-like lectin 7 mediates selective recognition of sialylated glycans expressed on campylobacter jejuni lipooligosaccharides. Infect Immun. (2006) 74:4133–41. doi: 10.1128/iai.02094-05, PMID: 16790787 PMC1489752

[19] Suenaga T Mori Y Suzutani T Arase H . Regulation of Siglec-7-mediated varicella-zoster virus infection of primary monocytes by cis-ligands. Biochem Biophys Res Commun. (2022) 613:41–6. doi: 10.1016/j.bbrc.2022.04.111, PMID: 35526487

[20] Suenaga T Mori Y Suzutani T Arase H . Siglec-7 mediates varicella-zoster virus infection by associating with glycoprotein B. Biochem Biophys Res Commun. (2022) 607:67–72. doi: 10.1016/j.bbrc.2022.03.060, PMID: 35367830

[21] Carluccio CD Amor TG Lenza MP Masi AA Abreu C Longo V . Molecular basis of siglec-7 recognition by neisseria meningitidis serogroup Y CPS: implications for immune evasion. JACS Au. (2025) 5:2257–69. doi: 10.1021/jacsau.5c00214, PMID: 40443894 PMC12117448

[22] Brunetta E Fogli M Varchetta S Bozzo L Hudspeth KL Marcenaro E . The decreased expression of Siglec-7 represents an early marker of dysfunctional natural killer–cell subsets associated with high levels of HIV-1 viremia. Blood. (2009) 114:3822–30. doi: 10.1182/blood-2009-06-226332, PMID: 19710502 PMC2773483

[23] Varchetta S Brunetta E Roberto A Mikulak J Hudspeth KL Mondelli MU . Engagement of siglec-7 receptor induces a pro-inflammatory response selectively in monocytes. PloS One. (2012) 7:e45821. doi: 10.1371/journal.pone.0045821, PMID: 23029261 PMC3461047

[24] Wisnovsky S Möckl L Malaker SA Pedram K Hess GT Riley NM . Genome-wide CRISPR screens reveal a specific ligand for the glycan-binding immune checkpoint receptor Siglec-7. Proc Natl Acad Sci. (2021) 118:e2015024118. doi: 10.1073/pnas.2015024118, PMID: 33495350 PMC7865165

[25] Yoshimura A Asahina Y Chang L-Y Angata T Tanaka H Kitajima K . Identification and functional characterization of a Siglec-7 counter-receptor on K562 cells. J Biol Chem. (2021) 296:100477. doi: 10.1016/j.jbc.2021.100477, PMID: 33640457 PMC8040268

[26] Stewart N Daly J Drummond-Guy O Krishnamoorthy V Stark JC Riley NM . The glyco-immune checkpoint receptor Siglec-7 interacts with T-cell ligands and regulates T-cell activation. J Biol Chem. (2024) 300(2):105579. doi: 10.1016/j.jbc.2023.105579, PMID: 38141764 PMC10831161

[27] Malhotra S Castilló J Bustamante M Vidal-Jordana A Castro Z Montalban X . SIGLEC1 and SIGLEC7 expression in circulating monocytes of patients with multiple sclerosis. Mult Scler J. (2012) 19:524–31. doi: 10.1177/1352458512458718, PMID: 22933622

[28] Dharmadhikari G Stolz K Hauke M Morgan NG Varki A de Koning E . Siglec-7 restores β-cell function and survival and reduces inflammation in pancreatic islets from patients with diabetes. Sci Rep. (2017) 7:45319. doi: 10.1038/srep45319, PMID: 28378743 PMC5381285

[29] Borges TJ Lima K Gassen RB Liu K Ganchiku Y Ribas GT . The inhibitory receptor Siglec-E controls antigen-presenting cell activation and T cell–mediated transplant rejection. Sci Transl Med. (2025) 17:eads2694. doi: 10.1126/scitranslmed.ads2694, PMID: 40333992 PMC12135932

[30] Mizrahi S Gibbs BF Karra L Ben-Zimra M Levi-Schaffer F . Siglec-7 is an inhibitory receptor on human mast cells and basophils. J Allergy Clin Immunol. (2014) 134:230–233.e3. doi: 10.1016/j.jaci.2014.03.031, PMID: 24810846

[31] Legrand F Landolina N Zaffran I Emeh RO Chen E Klion AD . Siglec-7 on peripheral blood eosinophils: Surface expression and function. Allergy. (2019) 74:1257–65. doi: 10.1111/all.13730, PMID: 30690753 PMC6661208

[32] Landolina N Zaffran I Smiljkovic D Serrano-Candelas E Schmiedel D Friedman S . Activation of Siglec-7 results in inhibition of *in vitro* and *in vivo* growth of human mast cell leukemia cells. Pharmacol Res. (2020) 158:104682. doi: 10.1016/j.phrs.2020.104682, PMID: 32035162

[33] Nakanishi M Tamagawa-Mineoka R Nishigaki H Arakawa Y Ohtsuka S Katoh N . Role of siglec-E in MC903-induced atopic dermatitis. Exp Dermatol. (2025) 34:e70064. doi: 10.1111/exd.70064, PMID: 39967561

[34] Liu H Zheng Y Zhang Y Li J Fernandes SM Zeng D . Immunosuppressive Siglec-E ligands on mouse aorta are up-regulated by LPS via NF-κB pathway. BioMed Pharmacother. (2020) 122:109760. doi: 10.1016/j.biopha.2019.109760, PMID: 31918287

[35] Liu H Li J Wu N She Y Luo Y Huang Y . Supplementing glucose intake reverses the inflammation induced by a high-fat diet by increasing the expression of siglec-E ligands on erythrocytes. Inflammation. (2024) 47:609–25. doi: 10.1007/s10753-023-01932-0, PMID: 38448631

[36] Zeng Z Li M Wang M Wu X Li Q Ning Q . Increased expression of Siglec-9 in chronic obstructive pulmonary disease. Sci Rep. (2017) 7:10116. doi: 10.1038/s41598-017-09120-5, PMID: 28860481 PMC5579055

[37] O’Sullivan JA Carroll DJ Cao Y Salicru AN Bochner BS . Leveraging Siglec-8 endocytic mechanisms to kill human eosinophils and Malignant mast cells. J Allergy Clin Immunol. (2018) 141:1774–1785.e7. doi: 10.1016/j.jaci.2017.06.028, PMID: 28734845 PMC6445644

[38] Chen L Patil S Barbon J Waire J Laroux FS McCarthy D . Agonistic anti-DCIR antibody inhibits ITAM-mediated inflammatory signaling and promotes immune resolution. JCI Insight. (2024) 9:e176064. doi: 10.1172/jci.insight.176064, PMID: 38781017 PMC11383175

[39] Hughes CS Moggridge S Müller T Sorensen PH Morin GB Krijgsveld J . Single-pot, solid-phase-enhanced sample preparation for proteomics experiments. Nat Protoc. (2019) 14:68–85. doi: 10.1038/s41596-018-0082-x, PMID: 30464214

[40] Hong S Yu C Rodrigues E Shi Y Chen H Wang P . Modulation of siglec-7 signaling via in situ-created high-affinity cis-ligands. ACS Cent Sci. (2021) 7:1338–46. doi: 10.1021/acscentsci.1c00064, PMID: 34471678 PMC8393205

[41] Nicoll G Avril T Lock K Furukawa K Bovin N Crocker PR . Ganglioside GD3 expression on target cells can modulate NK cell cytotoxicity via siglec-7-dependent and -independent mechanisms. Eur J Immunol. (2003) 33:1642–8. doi: 10.1002/eji.200323693, PMID: 12778482

[42] McCord KA Wang C Anhalt M Poon WW Gavin AL Wu P . Dissecting the ability of siglecs to antagonize fcγ Receptors. ACS Cent Sci. (2024) 10:315–30. doi: 10.1021/acscentsci.3c00969, PMID: 38435516 PMC10906256

[43] Pérez-Ferreros P Gaus K Goyette J . Tethered signaling in inhibitory immune receptors. Front Phys. (2019) 6:158. doi: 10.3389/fphy.2018.00158, PMID: 41816698

[44] Cullen PJ Steinberg F . To degrade or not to degrade: mechanisms and significance of endocytic recycling. Nat Rev Mol Cell Biol. (2018) 19:679–96. doi: 10.1038/s41580-018-0053-7, PMID: 30194414

[45] Orr SJ Morgan NM Buick RJ Boyd CR Elliott J Burrows JF . SOCS3 targets siglec 7 for proteasomal degradation and blocks siglec 7-mediated responses*. J Biol Chem. (2007) 282:3418–22. doi: 10.1074/jbc.c600216200, PMID: 17138568

[46] Kortmann J Brubaker SW Monack DM . Cutting edge: inflammasome activation in primary human macrophages is dependent on flagellin. J Immunol. (2015) 195:815–9. doi: 10.4049/jimmunol.1403100, PMID: 26109648 PMC4505955

[47] Bagaev AV Garaeva AY Lebedeva ES Pichugin AV Ataullakhanov RI Ataullakhanov FI . Elevated pre-activation basal level of nuclear NF-κB in native macrophages accelerates LPS-induced translocation of cytosolic NF-κB into the cell nucleus. Sci Rep. (2019) 9:4563. doi: 10.1038/s41598-018-36052-5, PMID: 30872589 PMC6418260

[48] Sharif O Bolshakov VN Raines S Newham P Perkins ND . Transcriptional profiling of the LPS induced NF-κB response in macrophages. BMC Immunol. (2007) 8:1. doi: 10.1186/1471-2172-8-1, PMID: 17222336 PMC1781469

[49] Şen B Balcı-Peynircioğlu B . Cellular models in autoinflammatory disease research. Clin Transl Immunol. (2024) 13:e1481. doi: 10.1002/cti2.1481, PMID: 38213819 PMC10784111

[50] Brzezicka KA Paulson JC . Impact of Siglecs on autoimmune diseases. Mol Asp Med. (2023) 90:101140. doi: 10.1016/j.mam.2022.101140, PMID: 36055802 PMC9905255

[51] Macauley MS Crocker PR Paulson JC . Siglec-mediated regulation of immune cell function in disease. Nat Rev Immunol. (2014) 14:653–66. doi: 10.1038/nri3737, PMID: 25234143 PMC4191907

[52] van Houtum EJ Valk AH Granado D Lok J van den Bogaard L Remkes N . Siglec-7 and Siglec-9 expression in primary triple negative and oestrogen receptor positive breast cancer and *in vitro* signalling. Clin Transl Immunol. (2024) 13:e1524. doi: 10.1002/cti2.1524, PMID: 39246414 PMC11378723

[53] Benthami H Zohair B Rezouki I Naji O Miyara K Ennachit S . Elevated Siglec-7 expression correlates with adverse clinicopathological, immunological, and therapeutic response signatures in breast cancer patients. Front Immunol. (2025) 16:1573365. doi: 10.3389/fimmu.2025.1573365, PMID: 40547037 PMC12179189

[54] Jiang K-Y Qi L-L Kang F-B Wang L . The intriguing roles of Siglec family members in the tumor microenvironment. biomark Res. (2022) 10:22. doi: 10.1186/s40364-022-00369-1, PMID: 35418152 PMC9008986

[55] Ibarlucea-Benitez I Weitzenfeld P Smith P Ravetch JV . Siglecs-7/9 function as inhibitory immune checkpoints *in vivo* and can be targeted to enhance therapeutic antitumor immunity. Proc Natl Acad Sci. (2021) 118:e2107424118. doi: 10.1073/pnas.2107424118, PMID: 34155121 PMC8256000

[56] Wang C Hou Y Zak J Zheng Q McCord KA Wu M . Reshaping the tumor microenvironment by degrading glycoimmune checkpoints Siglec-7 and -9. bioRxiv. (2024). doi: 10.1101/2024.10.11.617879. 2024.10.11.617879. PMID: 39416090 PMC11483058

[57] Bordoloi D Kulkarni AJ Adeniji OS Pampena MB Bhojnagarwala PS Zhao S . Siglec-7 glyco-immune binding mAbs or NK cell engager biologics induce potent antitumor immunity against ovarian cancers. Sci Adv. (2023) 9:eadh4379. doi: 10.1126/sciadv.adh4379, PMID: 37910620 PMC10619929

[58] Szabó E Faragó A Bodor G Gémes N Puskás LG Kovács L . Identification of immune subsets with distinct lectin binding signatures using multi-parameter flow cytometry: correlations with disease activity in systemic lupus erythematosus. Front Immunol. (2024) 15:1380481. doi: 10.3389/fimmu.2024.1380481, PMID: 38774868 PMC11106380

[59] Amith SR Jayanth P Franchuk S Finlay T Seyrantepe V Beyaert R . Neu1 desialylation of sialyl α-2,3-linked β-galactosyl residues of TOLL-like receptor 4 is essential for receptor activation and cellular signaling. Cell Signal. (2010) 22:314–24. doi: 10.1016/j.cellsig.2009.09.038, PMID: 19796680

[60] Miralda I Samanas NB Seo AJ Foronda JS Sachen J Hui Y . Siglec-9 is an inhibitory receptor on human mast cells *in vitro*. J Allergy Clin Immunol. (2023) 152:711–724.e14. doi: 10.1016/j.jaci.2023.04.007, PMID: 37100120 PMC10524464

[61] Shan D Press OW . Constitutive endocytosis and degradation of CD22 by human B cells. J Immunol (Baltim Md : 1950). (1995) 154:4466–75. doi: 10.4049/jimmunol.154.9.4466, PMID: 7722303

[62] Tateno H Li H Schur MJ Bovin N Crocker PR Wakarchuk WW . Distinct endocytic mechanisms of CD22 (Siglec-2) and siglec-F reflect roles in cell signaling and innate immunity. Mol Cell Biol. (2007) 27:5699–710. doi: 10.1128/mcb.00383-07, PMID: 17562860 PMC1952126

[63] Kawai T Ikegawa M Ori D Akira S . Decoding Toll-like receptors: Recent insights and perspectives in innate immunity. Immunity. (2024) 57:649–73. doi: 10.1016/j.immuni.2024.03.004, PMID: 38599164

[64] Paluch C Santos AM Anzilotti C Cornall RJ Davis SJ . Immune checkpoints as therapeutic targets in autoimmunity. Front Immunol. (2018) 9:2306. doi: 10.3389/fimmu.2018.02306, PMID: 30349540 PMC6186808

[65] Kuchroo JR Goldman N Sharpe AH . PD-1, BTLA and TIGIT as therapeutic targets for rheumatic disease. Nat Rev Rheumatol. (2026) 22, 89–104. doi: 10.1038/s41584-025-01296-9, PMID: 41331129

[66] Zhuang Q Liu Q Divito SJ Zeng Q Yatim KM Hughes AD . Graft-infiltrating host dendritic cells play a key role in organ transplant rejection. Nat Commun. (2016) 7:12623. doi: 10.1038/ncomms12623, PMID: 27554168 PMC4999515

[67] Ochando J Ordikhani F Boros P Jordan S . The innate immune response to allotransplants: mechanisms and therapeutic potentials. Cell Mol Immunol. (2019) 16:350–6. doi: 10.1038/s41423-019-0216-2, PMID: 30804476 PMC6462017

[68] Gollmann-Tepeköylü C Graber M Pölzl L Nägele F Moling R Esser H . Toll-like receptor 3 mediates ischaemia/reperfusion injury after cardiac transplantation. Eur J Cardio-Thorac Surg. (2020) 57:826–35. doi: 10.1093/ejcts/ezz383, PMID: 32040169

[69] Zhao J Huang X Mcleod P Jiang J Liu W Haig A . Toll-like receptor 3 is an endogenous sensor of cell death and a potential target for induction of long-term cardiac transplant survival. Am J Transplant. (2021) 21:3268–79. doi: 10.1111/ajt.16584, PMID: 33784431

[70] Redondo N Rodríguez-Goncer I Parra P Ruiz-Merlo T López-Medrano F González E . Influence of single-nucleotide polymorphisms in TLR3 (rs3775291) and TLR9 (rs352139) on the risk of CMV infection in kidney transplant recipients. Front Immunol. (2022) 13:929995. doi: 10.3389/fimmu.2022.929995, PMID: 35967300 PMC9374175

[71] Redondo N Navarro D Aguado JM Fernández-Ruiz M . Human genetic polymorphisms and risk of viral infection after solid organ transplantation. Transplant Rev. (2022) 36:100669. doi: 10.1016/j.trre.2021.100669, PMID: 34688126

[72] Cohen GS Kallarakal MA Jayaraman S Ibukun FI Tong KP Orzolek LD . Transplantation elicits a clonally diverse CD8+ T cell response that is comprised of potent CD43+ effectors. Cell Rep. (2023) 42:112993. doi: 10.1016/j.celrep.2023.112993, PMID: 37590141 PMC10727118

[73] Pinho SS Alves I Gaifem J Rabinovich GA . Immune regulatory networks coordinated by glycans and glycan-binding proteins in autoimmunity and infection. Cell Mol Immunol. (2023) 20:1101–13. doi: 10.1038/s41423-023-01074-1, PMID: 37582971 PMC10541879

[74] Siegel CH Sammaritano LR . Systemic lupus erythematosus. JAMA. (2024) 331:1480–91. doi: 10.1001/jama.2024.2315, PMID: 38587826

[75] Flores R Zhang P Wu W Wang X Ye P Zheng P . Siglec genes confer resistance to systemic lupus erythematosus in humans and mice. Cell Mol Immunol. (2019) 16:154–64. doi: 10.1038/cmi.2017.160, PMID: 29503442 PMC6355849

