# Supplementary figures and images for: Siglec-7 inhibits TLR3-induced pro-inflammatory cytokine production from human monocytes and macrophages
Source: Front Immunol. 2026 Mar 17;17:1764343. doi: 10.3389/fimmu.2026.1764343 (PMC13035513; doi:10.3389/fimmu.2026.1764343)

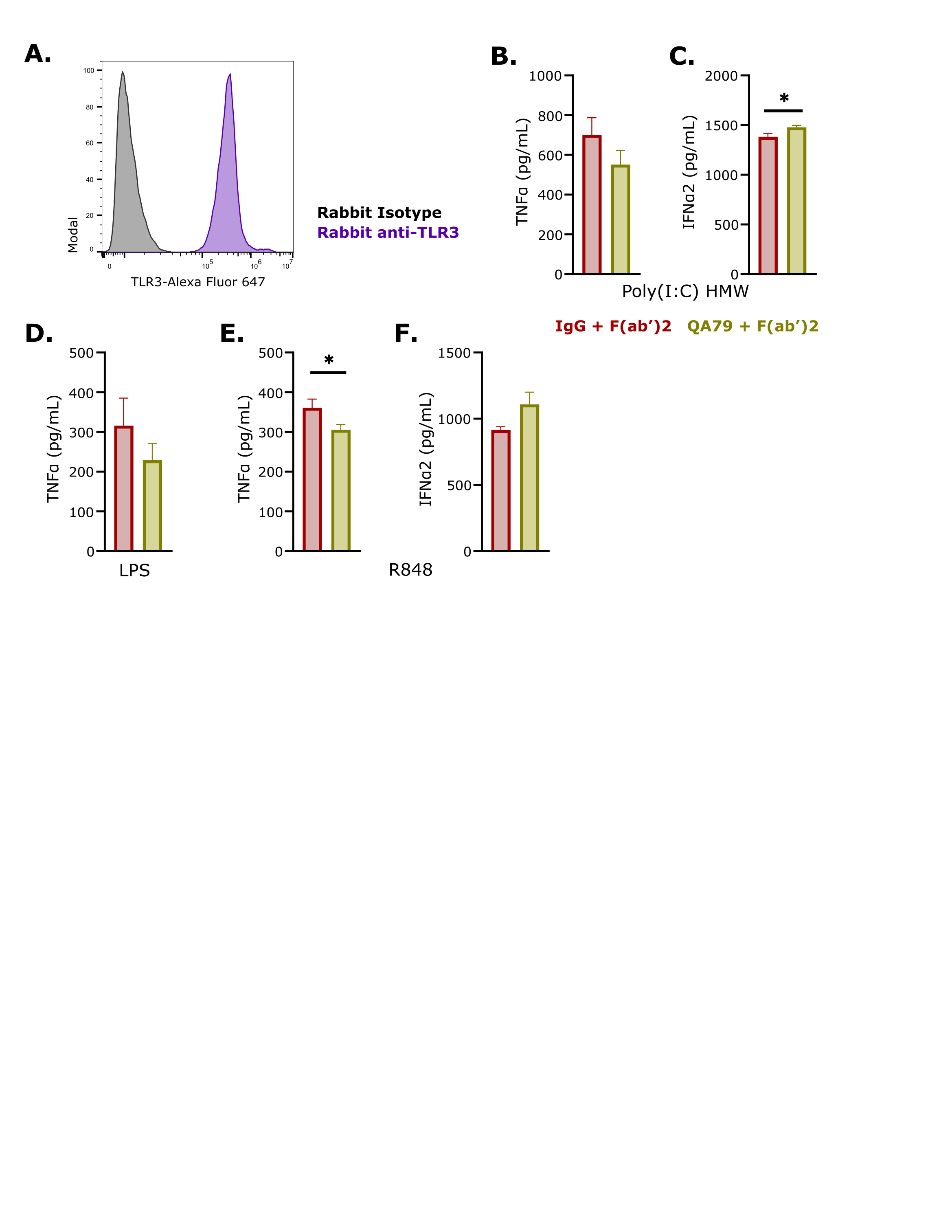

Supplement: Supplementary Figure 1 — Siglec-7 agonism of U937 cells inhibits proinflammatory cytokines from endosomal TLRs. (A) Representative flow cytometry histogram of TLR3 staining in U937 cells. Black: Isotype, Purple: TLR3 (n=2). TNFα (B) or IFNα2 (C) ELISA from U937 cells preincubated with 10 µg/mL IgG or QA79 for 30 minutes on ice prior to treating with 2 µg/mL F(ab’)2 and 1.25 µg/mL Poly(I:C) HMW for 24 hours. Data are mean ± SEM, and statistics are a Student T-test; *p<0.05 (n=3 experiments ran in triplicate). (D) TNFα ELISA from U937 cells preincubated with 10 µg/mL IgG or QA79 for 30 minutes on ice prior to treating with 2 µg/mL F(ab’)2 and 2 ng/mL LPS for 24 hours. Data are mean ± SEM, and statistics are a Student T-test (n=2 experiments ran in triplicate). TNFα (E) or IFNα2 (F) ELISA from U937 cells preincubated with 10 µg/mL IgG or QA79 for 30 minutes on ice prior to treating with 2 µg/mL F(ab’)2 and 2.5 µg/mL R848 for 24 hours. Data are mean ± SEM, and statistics are a Student T-test; *p<0.05 (n=3 experiments ran in triplicate). [file Image1.tiff]

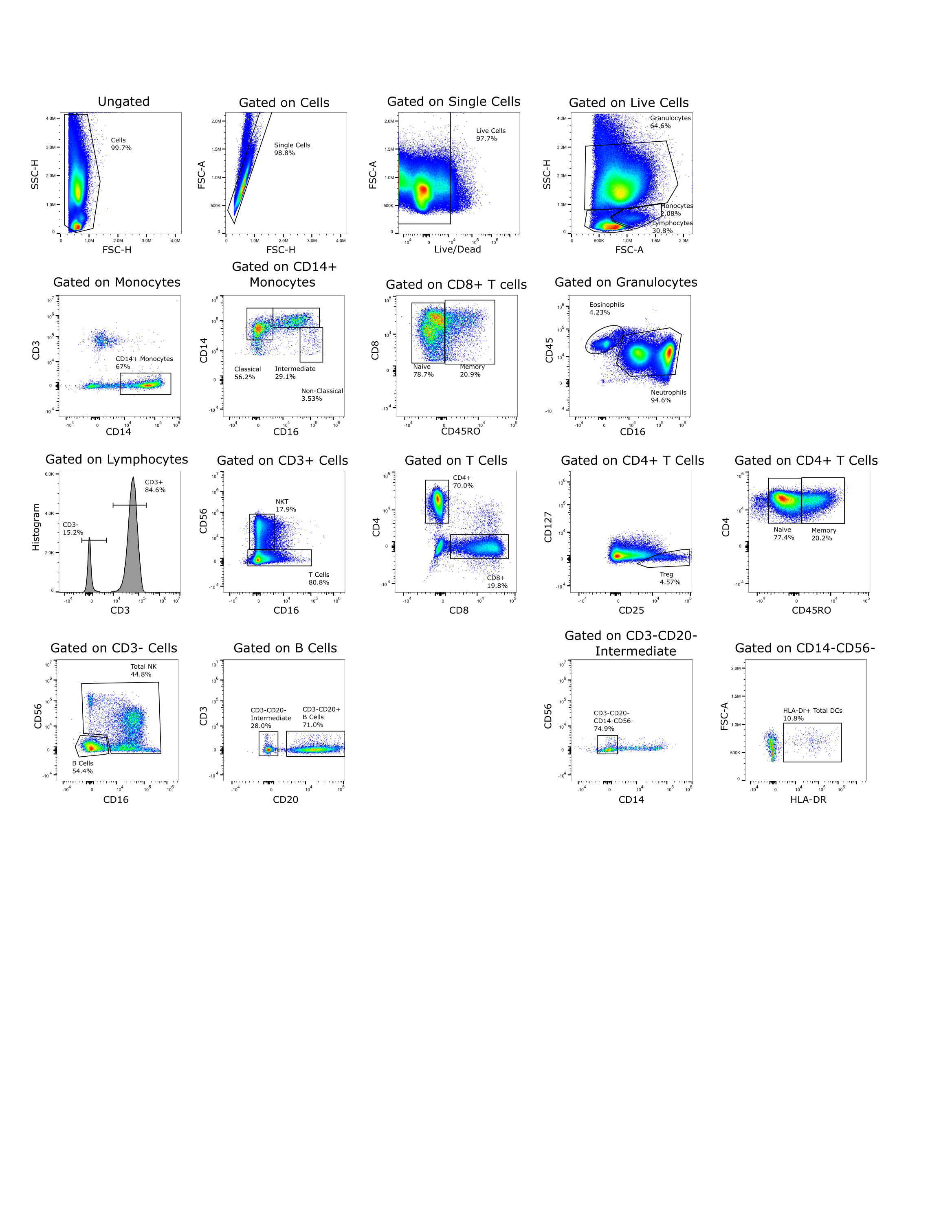

Supplement: Supplementary Figure 2 — Gating strategy of human whole blood Siglec-7 immunophenotyping. [file Image2.tiff]

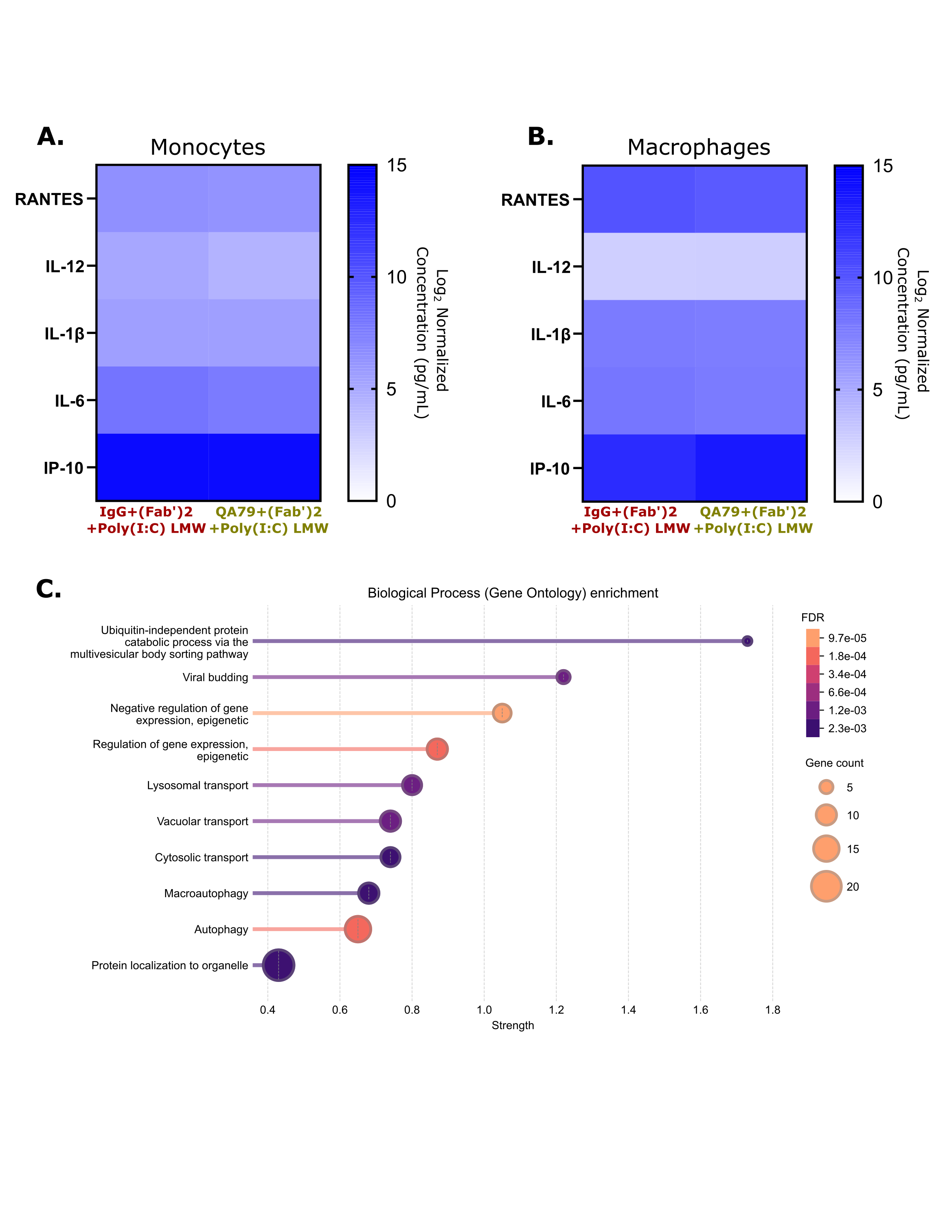

Supplement: Supplementary Figure 3 — Siglec-7 agonism indued minimal cytokine changes in primary cells. MSD multiplex assay profiling additional cytokines known to be downstream of TLR3 activation from primary human monocytes (A) or primary human macrophages (B) preincubated with 20 µg/mL IgG or QA79 for 30 minutes on ice prior to treating with 2 µg/mL F(ab’)2 and 1.25 µg/mL Poly(I:C) LMW for 24 hours. (n=1 donor ran in triplicate). (C) Protein Set Enrichment analysis of proteins significantly altered by a 3 hour stimulation with plate bound QA79 compared to IgG control in primary macrophages. (n=3 donors). [file Image3.tiff]
